# Hormone Receptor Expression in Primary and Recurrent High-Grade Serous Ovarian Cancer and Its Implications in Early Maintenance Treatment

**DOI:** 10.3390/ijms232214242

**Published:** 2022-11-17

**Authors:** Marcus Vetter, Sylvia Stadlmann, Evelyne Bischof, Elena Laura Georgescu Margarint, Andreas Schötzau, Gad Singer, Viola Heinzelmann-Schwarz, Céline Montavon

**Affiliations:** 1 Gynecologic Cancer Center, University Basel, Spitalstrasse 21, 4031 Basel, Switzerland; 2 Department of Pathology, Kantonsspital Baden AG, Im Ergel 1, 5404 Baden, Switzerland; 3 Department of Basic and Clinical Medicine, Shanghai University of Medicine and Health Sciences, Shanghai 201318, China; 4 Department of Medical Oncology, Renji Hospital, School of Medicine, Shanghai Jiao Tong University, Shanghai 200240, China; 5 Shanghai East International Medical Center, Shanghai 200120, China; 6 Ovarian Cancer Research, Department of Biomedicine, University of Basel, 4031 Basel, Switzerland

**Keywords:** high-grade serous ovarian cancer, HGSOC, hormone receptors, ER, PR, relapsed ovarian cancer

## Abstract

Endocrine therapy is an effective treatment for low-grade serous ovarian cancer. However, the role of estrogen and progesterone receptors as biomarkers for high-grade serous ovarian cancer (HGSOC) is yet to be elucidated because not all estrogen and progesterone receptor-positive tumors benefit from anti-estrogen therapy. The degree of expression is presumed to play a vital role; however, that role is not well-defined in ovarian cancer. We aimed to determine the role of estrogen and progesterone receptor expression in primary and paired relapsed HGSOC. In this study, primary and matched relapsed tumor samples were collected from 80 patients with International Federation of Gynecology and Obstetrics Stage II–IV HGSOC. Tissue microarray was conducted and immunohistochemistry for estrogen and progesterone receptor expression was performed. Two independent pathologists performed the tissue microarray analysis with the Immunoreactive Score and Allred Total score. In the paired analysis, no significant difference in estrogen receptor expression was observed. However, progesterone receptor expression was significantly lower in patients with recurrent platinum-sensitive HGSOC. We conclude that anti-estrogen therapy targeting estrogen receptor positive HGSOC could be administered in primary and relapsed settings. The use of endocrine maintenance with an aromatase inhibitor in patients with estrogen receptor positive HGSOC needs to be further evaluated and validated in a randomized controlled trial.

## 1. Introduction

Globally, ovarian cancer accounts for an estimated 239,000 new cases and 152,000 deaths annually [1]. The prognosis of ovarian cancer remains poor, with a 5-year overall survival (OS) rate of 30–40% [2]. The stage and the histopathological subtype affect the prognosis, with worse outcomes observed in cases of clear cell and high-grade serous ovarian cancer (HGSOC) [3]. HGSOC is the most common subtype of ovarian cancer, accounting for about 60–70% of all ovarian cancers, and has a poor prognosis [4]. In the last decade, progress has been made in surgical [5], systemic [6], and targeted therapeutic interventions [7,8,9], as well as neo-adjuvant and/or adjuvant management with endocrine agents, such as aromatase inhibitors, fulvestrant, gonadotropin-releasing hormone analogues, and tamoxifen [10]. The aim of endocrine therapy is to delay treatment with toxic chemotherapies and extend the time to next treatment, which has become an important endpoint in studies with poly adenosine diphosphate ribose polymerase-inhibitors [11]. Thus far, poly adenosine diphosphate ribose polymerase-inhibitors and bevacizumab are the most commonly used drugs in primary and relapse maintenance strategies [9,12].

Anti-estrogen drugs, such as aromatase inhibitors, have been demonstrated to have certain benefits in estrogen receptor positive ovarian cancer patients [13]. A recently published randomized phase III study, which included over 230 patients, compared the role of tamoxifen versus chemotherapy in platinum-resistant relapsed ovarian cancer patients. Although progression-free survival improved in the chemotherapy arm, no difference in overall survival was observed; instead, impaired quality of life was observed in patients [14]. This trial emphasizes the potential role of anti-estrogen treatment in ovarian cancer, which, thus far, may have been underestimated.

The role of estrogen and progesterone receptor expression, along with concordance of hormonal receptors, as predictive biomarkers in primary and relapsed HGSOC remains debatable and controversial. Many clinicians administer endocrine therapy in relapsed settings based on a smaller phase II trial or retrospective case series [15,16,17]. A recently published prospective study, the PARAGON trial, which examined the role of the aromatase inhibitor anastrozole in relapsed gynecologic cancers, reported a disease control rate of 36% at 3 months and demonstrated a stable quality of life for patients treated with aromatase inhibitors [18,19]. A recent comprehensive review of the available evidence revealed that most of the related trials were small and not comparable, and estrogen and progesterone receptor expression in the cohorts was not examined. Since 1982, more than 50 trials with extremely heterogeneous patient populations have been published, reporting a clinical benefit rate of 0–50% [13]. The most common drugs used were tamoxifen and letrozole. To date, endocrine therapy has shown a significantly positive impact in low-grade serous ovarian cancer [20]. The largest retrospective subset analysis demonstrated an increase in progression-free survival by almost 40 months when endocrine maintenance therapy was administered after primary surgery and first-line chemotherapy [20]. The current European Society of Medical Oncology/European Society of Gynaecological Oncology and National Comprehensive Cancer Network (NCCN) guidelines support the use of endocrine agents as an option for relapsed serous ovarian cancer [21,22]. Ongoing studies are currently investigating the factors that influence the efficacy of anti-estrogen, such as histological subtype (low-grade serous ovarian cancer), genetic signatures, high levels of estrogen and progesterone receptor expression, long treatment intervals (late recurrence beyond 24 months), and addition of aromatase inhibitors (versus other anti-estrogenic agents) [20,23].

The early use of maintenance endocrine therapy, following primary debulking surgery and first-line chemotherapy, including targeted therapy with poly adenosine diphosphate ribose polymerase or vascular endothelial growth factor inhibitors, is yet to be elucidated. In a recent study, we demonstrated that primary maintenance of HGSOC with letrozole may be beneficial after primary surgery and chemotherapy [24]. Currently, we have initiated an international collaborative trial that evaluates the role of letrozole in the primary maintenance setting called maintenance therapy with aromatase inhibitor in epithelial ovarian cancer; we are conducting a randomized, double-blinded, placebo-controlled, multi-center phase III trial (ENGOT-ov54/Swiss-GO-2/MATAO), ClinicalTrials.gov, accessed on 26 January 2020 (Identifier: NCT04111978). Compared with other maintenance therapies, an early endocrine strategy has the advantage of better tolerability of letrozole, thus improving the overall quality of life and cost-effectiveness while also improving the time to next treatment [25].

The prognostic role of estrogen and progesterone receptor expression in ovarian cancer is well established. A large consortia study by Sieh et al. examined the prognostic role of these receptors in more than 2900 patients with primary ovarian cancer, including over 1700 patients with HGSOC, and reported that estrogen receptor was expressed in >80% of HGSOC cases [26]. In the correlation analysis, however, compared to low estrogen receptor expression, high expression was not associated with improved disease-specific survival (HR 1.05; 0.89–1.24; *p* = 0.56). However, a high progesterone receptor expression was independently associated with improved disease-specific survival in HGSOC patients (HR 0.71; 0.55–0.91; *p* = 0.0080). This potentially crucial predictive and prognostic value of progesterone receptor expression in HGSOC needs to be assessed in further clinical trials [23]. Furthermore, a recent study conducted in China reported a discordance in estrogen and progesterone receptor expression (significantly lower or higher expression in the relapsed sample) in 34% and 12% of patients with paired primary and recurrent HGSOC, respectively [27]. The authors demonstrated no impact of receptor discordance on the outcomes of HGSOC patients.

Therefore, our study focused on the role of estrogen and progesterone receptor expression in a Swiss cohort comprising primary and paired relapsed platinum-sensitive and platinum-resistant HGSOC patients. We investigated (1) the expression of estrogen and progesterone receptors in the HGSOC cohort, (2) the concordance of estrogen and progesterone receptor expression in primary and relapsed HGSOC patients, and (3) the integration of the expression pattern of estrogen and progesterone within a decision tree for or against anti-estrogen treatment.

## 2. Results

### 2.1. Clinicopathological Patient Characteristics

Tissue samples obtained from 80 patients were included in this study. All patients underwent standard debulking surgery for HGSOC, followed by six cycles of platinum-based chemotherapy (completed by 73 patients [91.3%]; Table 1). The median age of the patients was 59 years (range, 20–77 years), and most patients had an advanced-stage disease (FIGO stage III/IV, 96.3%). In recurrent tumors, the majority (57/80, 71.3%) were platinum-sensitive; however, 23 of 80 tumors were platinum-resistant (28.7%).

### 2.2. Evaluating Estrogen and Progesterone Receptor Expression in Primary Chemotherapy-Sensitive and -Resistant HGSOC Patients Using Two Independent Immunohistochemistry Scoring Systems

Estrogen receptor positivity was identified in 26% (19 of 73) and 35.6% (26 of 73) of the samples as evaluated by the Immunoreactive Score and the Allred Total score, respectively. The corresponding values for progesterone receptor positivity assessed by the two scoring systems were 27.5% (19 of 69) and 30.4% (21 of 69) of the samples, respectively.

In the subgroup of chemotherapy-sensitive primary HGSOC (*n* = 52 [71.2%]), 23.1% (12 of 52) and 32.7% (17 of 52) of the samples were estrogen receptor positive based on the Immunoreactive Score and the Allred Total score, respectively. Progesterone receptor expression was positive in 29.2% (14 of 48) and 31.3% (15 of 48) of the samples based on the Immunoreactive Score and the Allred Total score, respectively (Table 2a,b). Estrogen and progesterone receptor staining was unsuccessful in five and nine samples, respectively.

In the smaller subgroup of primary chemotherapy-resistant HGSOC (*n* = 21 [26.9%]), estrogen receptor expression was positive in 33.3% (7 of 21) and 42.9% (9 of 21) of the samples based on the Immunoreactive Score and the Allred Total score, respectively. Progesterone receptor expression was positive in 23.8% (5 of 21) and 28.6% (6 of 21) of the samples based on the Immunoreactive Score and the Allred Total score, respectively (Table 2a,b). Estrogen and progesterone receptor staining was unsuccessful in two samples each.

### 2.3. Estrogen and Progesterone Receptor Expression in Recurrent Chemotherapy-Sensitive and Resistant HGSOC

To quantify estrogen and progesterone receptor expression in recurrent tumor samples, the same independent scoring systems were used. In the recurrent tumor samples (sensible and resistant tumors) 18.3% (13 of 71) and 39.4% (28 of 71) of the samples were estrogen receptor positive based on the Immunoreactive Score and the Allred Total score, respectively. Progesterone receptor positivity was identified in 13.8% (10 of 72) and 20.83% (15 of 72) of the samples based on the Immunoreactive Score and the Allred Total score, respectively (Table 2a,b).

In the chemotherapy-sensitive recurrent HGSOC subgroup (*n* = 50 [70.42%]), estrogen receptor expression positive in 18% (9 of 50) and 34% (17 of 50) of the samples based on the Immunoreactive Score and the Allred Total score, respectively. Progesterone receptor positivity was identified in 13.5% (7 of 52) and 19.2% (10 of 52) of the samples based on the Immunoreactive Score and the Allred Total score, respectively. Progesterone receptor staining was unsuccessful in two cases.

In the chemotherapy-resistant recurrent HGSOC (*n* = 21 [29.58%]), 19% (4 of 21) and 52.4% (11 of 21) of the samples were estrogen receptor positive based on the Immunoreactive Score and the Allred Total score, respectively, whereas progesterone receptor expression was positive in 15% (3 of 20) and 25% (5 of 20 samples) of the samples based on the Immunoreactive Score and the Allred Total score, respectively (Table 2a,b).

### 2.4. Correlation of Estrogen and Progesterone Receptor Expression in Paired Primary and Recurrent HGSOC

#### 2.4.1. Estrogen Receptor Expression

**Figure 1:**
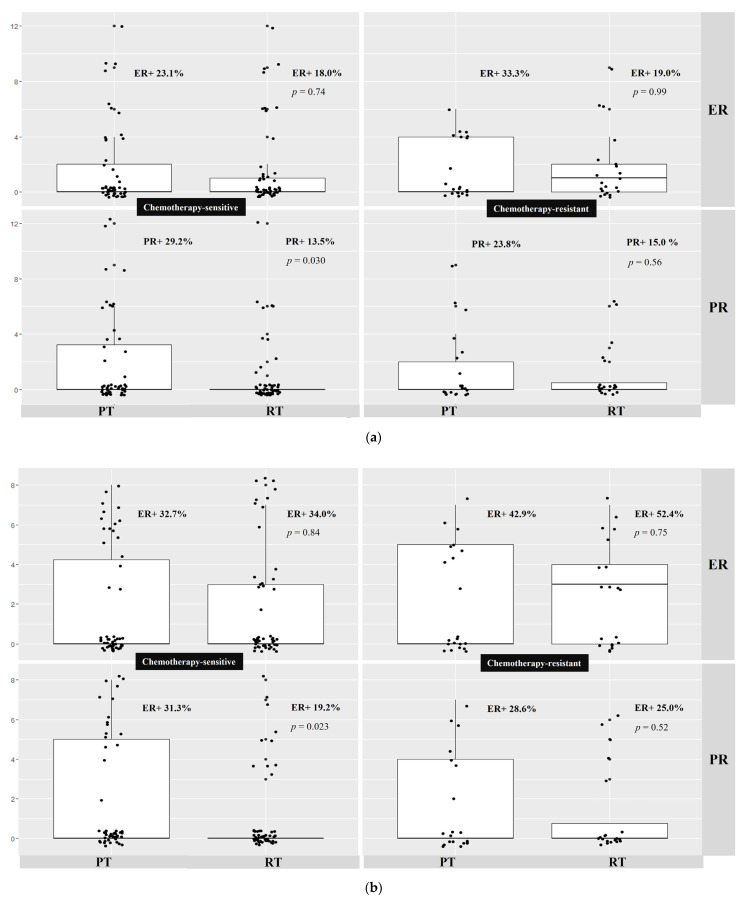
(**a**) Association of estrogen and progesterone receptor immunoexpression between primary and recurrent tumors (Immuno-reactive Score System); (**b**) Association of estrogen and progesterone receptor immunoexpression between primary and recurrent tumors (Allred Total Score System). (**a**) shows the distribution of Immunoreactive Score for estrogen and progesterone receptor expression in primary and paired recurrent HGSOC. ER, estrogen receptor; PR, progesterone receptor; PT, primary tumor; RT, paired recurrent tumor; HGSOC, high-grade serous ovarian cancer; IRS, Immunoreactive Score. There was a statistically significant lower PR expression in chemotherapy-sensitive-matched primary and recurrent HGSOC. (**b**) shows the distribution of Allred Total Score for estrogen and progesterone receptor expression in primary and paired recurrent HGSOC. ER, estrogen receptor; PR, progesterone receptor; PT, primary tumor; RT, paired recurrent tumor; HGSOC, high-grade serous ovarian cancer; ATS, Allred Total score. Progesterone receptor expression was statistically significantly lower in chemotherapy-sensitive-matched primary and recurrent HGSOC.

There was no significant difference in estrogen receptor expression between the paired primary and recurrent samples using the two independent immunohistochemistry scoring systems. Specifically, no difference was observed in estrogen receptor expression between chemotherapy-sensitive primary and relapsed HGSOC patient samples (Immunoreactive Score: 23.1% vs. 18%, *p* = 0.74; Allred Total score: 32.7 vs. 34%, *p* = 0.84). No difference in estrogen receptor expression was observed between chemotherapy-resistant primary and relapsed HGSOC patient samples (Immunoreactive Score: 33.3% vs. 19%, *p* = 0.99; Allred Total score: 42.9% vs. 52.4%, *p* = 0.75). Further, no difference was observed in estrogen receptor expression between primary-sensitive and primary-resistant HGSOC. Additionally, no difference was found between the recurrent sensitive and recurrent resistant samples (Table 2a,b, Figure 1a,b).

#### 2.4.2. Progesterone Receptor Expression

Both immunohistochemistry scoring systems revealed significantly lower progesterone receptor expression in primary chemotherapy-sensitive compared to paired chemotherapy-sensitive recurrent HGSOC patient samples (Immunoreactive Score: 29.2% vs. 13.5%, *p* = 0.030; Allred Total score: 31.3% vs. 19.2%, *p* = 0.023). There was no difference observed in PR expression in primary chemotherapy-resistant and paired chemotherapy-resistant recurrent HGSOC patient samples (Immunoreactive Score: 23.8% vs. 15%, *p* = 0.56; Allred Total score: 28.6% vs. 25% *p* = 0.52). Moreover, no difference in progesterone receptor expression was observed between the primary-sensitive and primary-resistant HGSOC patient samples. Additionally, no difference in progesterone receptor expression was found between the recurrent sensitive and recurrent resistant samples (Table 2a,b, Figure 1a,b).

## 3. Discussion

### 3.1. Summary of Main Results

Using two independent immunohistochemical scoring systems, we found that estrogen receptor expression was not significantly different between primary and paired relapsed HGSOC patients; this finding was independent of the tumor’s sensitivity to platinum-based chemotherapy. Thus, the results suggest that endocrine therapy should be considered as a potential active agent in recurrent and platinum-resistant cancer.

### 3.2. Results in the Context of Published Literature

The prognosis of HGSOC remains poor, with a 5-year overall survival rate of 40–50%. Despite recent advances in treatment options, the long-term outcome of patients with advanced disease remains unfavorable. HGSOC patients significantly benefited from primary maintenance therapy with poly adenosine diphosphate ribose polymerase-inhibitors [25,28]. Additionally, acquired resistance to PARP inhibitors has been reported in an increasing population of patients [29]. Therefore, alternative and efficient treatment options are urgently required, especially in maintenance settings [30].

In a recent study, we demonstrated that adjuvant letrozole following primary debulking surgery and chemotherapy may significantly improve outcomes in HGSOC patients [24]. Anti-estrogen therapy during the early course of the disease was shown to substantially improve prognosis; therefore, evaluating estrogen and progesterone receptor expression is necessary for selecting patients who would benefit highly from this therapy, and for identifying biomarkers to detect early recurrence. Although more than 80% of HGSOC cases are estrogen receptor positive [26], the effects of anti-estrogen drugs on clinical outcomes are inconsistent, and studies have indicated that these treatments are beneficial for only a small proportion of patients [13]. Low progesterone levels induce less sensitivity of the tumors to chemotherapy. In sharp contrast with breast tissue where progesterone acts in concert with estrogen to promote proliferative gene programs, in the uterus progesterone hinders estrogen-driven growth. Moreover, progesterone shields the ovary from neoplastic transformation [31]. Recent findings demonstrate that the progesterone receptor modulates estrogen receptor chromatin binding to antagonize estrogen action. Selective PR modulators/antagonists can increase responses to antiestrogens, suggesting that therapies directed at ER and PR in ER+/PR+ breast cancers should be further investigated [32].

Several clinical guidelines recommend endocrine therapy in relapsed ovarian cancer based on estrogen and progesterone receptor expression levels in primary HGSOC patients [30]. There are no specific recommendations for their use in maintenance settings after front-line treatments. Therefore, this study analyzed the level of estrogen and progesterone expression in primary and recurrent HGSOC.

Our results are in contrast to recently reported data from a Chinese cohort (*n* = 107, HGSOC), where higher estrogen receptor expression (72.9%) was found in relapsed tumors compared to primary tumors (67), with a non-significant discordance rate of 34% (*p* = 0.324) [27]. A few possible reasons for the much lower numbers (18.3–39.4% of ER+ patients) might be the use of older tissue, the type of antibody utilized in the methodology, and different methods of analysis. A larger European consortia study confirmed these observations [26]. However, in our cohort we observed a slight decrease in estrogen receptor expression in the recurrent setting. It is noteworthy that none of these observations were statistically significant.

The clinical features and outcomes of HGSOC may differ between Western and Asian populations. A recently published study compared Caucasian and Asian HGSOC patients and reported better outcomes in clinical trials in Asian populations after adjusting for prognostic factors. The Asians enrolled in clinical trials were younger, had better performance statuses, earlier-stage disease, and a larger number of clear cell and mucinous tumors. After adjusting for these prognostic factors, the Asians had a better survival rate than the Caucasians [33].

Although the prognostic value of progesterone receptor expression has been observed in primary and recurrent disease, the available data remain controversial. A recent meta-analysis demonstrated that higher progesterone receptor expression led to better outcomes for ovarian cancer patients; suggesting that higher PR expression is a favorable prognostic marker [34]. We demonstrated a significant difference in progesterone receptor expression between primary and paired recurrent HGSOC samples, with lower expression in the latter. However, this effect was observed in platinum-sensitive tumors alone. There was no significant reduction in progesterone receptor expression among patients with platinum-resistant disease. As a limitation of our study, integration of the expression pattern of ER and PR within a decision tree for/against anti-estrogen treatment was not suitable since it is still not clear which patients benefit from AI treatment.

In contrast, Feng et al. reported that in a Chinese cohort, the level of progesterone receptor expression remained low in both primary and recurrent specimens (9.3% and 6.7%, respectively) [27]; whereas the level of progesterone receptor was much higher in our cohort (IRS primary vs. relapsed: 27.2% vs. 13.8%; ATS 30.4% vs. 20.8%). Moreover, we did not observe significant differences in progesterone receptor expression between platinum-sensitive and -resistant primary samples and between and platinum-sensitive and -resistant relapsed HGSOC patients. Overall, our observations strongly support the early use of endocrine therapy in ovarian cancer patients owing to decreased and/or lost progesterone receptor expression during disease progression.

### 3.3. Implications for Practice and Future Research

The loss of progesterone receptor expression in relapsed platinum-sensitive samples may be an important factor for further therapeutic decisions. The progesterone receptor is an intracellular polypeptide that translocates into the nucleus upon binding to progesterone, where it regulates the expression of a specific set of genes [35]. Activation of progestational signaling can suppress ovulation, antagonize the growth-promoting effect of estrogen, and regulate ovarian cancer cell proliferation and apoptosis [36]. Although the progesterone receptor is not well-established as a therapeutic target in HGSOC, mifepristone (RU486), a progesterone receptor antagonist, was demonstrated to have a response rate of 26.5% in cisplatin- and paclitaxel-resistant ovarian cancer [37].

Future studies should also consider the relationship between different expression profiles of estrogen and progesterone receptor positivity and negativity in ovarian cancer. Feng et al. [27] demonstrated that HGSOC patients with estrogen receptor positive and progesterone and androgen receptor negative tumors had the worst outcomes and concluded that this subgroup may require aggressive therapy. Based on our observations, early maintenance therapy with aromatase inhibitors is recommended for this subgroup of patients, which is also supported by previous studies demonstrating a therapeutic effect of receptor distribution and expression levels in patients with metastatic estrogen and progesterone receptor positive breast cancers [38,39].

## 4. Materials and Methods

### 4.1. Patient Cohort

All procedures performed in this study involving human participants were in accordance with the ethical standards of the institutional and/or national research committee and with the 1964 Helsinki Declaration and its ethical standards. The requirement for informed consent was waived due to the retrospective nature of this study and because data accession was anonymous. The statement concerning the clinical data collection and ethical considerations can be found in previous publications [40,41].

Primary and matched tumor samples were collected from 80 patients with advanced International Federation of Gynecology and Obstetrics (FIGO) Stage II–IV (96.3% patients were of FIGO Stage III–IV) high-grade serous ovarian adenocarcinomas, over a period of 17 years (from 1985 to 2003) and arranged in a tissue microarray following a pathological review. The patients’ characteristics are presented in Table 1.

Tissues were collected from the Institute of Pathology of the University of Basel and the Cantonal Hospitals of Baden, Liestal, and St. Gallen, according to the guidelines of the Institutional Review Boards of the participating institutions, and specific guidelines for the Institute of Pathology of the University Hospital Basel were used for the tissue microarray construction.

Construction and subsequent studies using the tissue microarray were supported by the Swiss Cancer League (Oncosuisse) Grant number OCS 01506-02-2004. All clinical data related to the respective tissue samples were collected from medical chart reviews of patients diagnosed with HGSOC between 1985 and 2003. The time of recurrence was defined as the time of a biochemical and clinical relapse with elevation of CA-125 levels, along with confirmed response evaluation criteria in solid tumors in the radiological examination and/or during a secondary surgery. Cancers with a recurrence time of ≤6 months after completion of platinum-based chemotherapy were defined as platinum-resistant, and cancers with a recurrence time of >6 months were platinum-sensitive, in accordance with the Gynecological Cancer Intergroup definition. This definition is arbitrary and has recently been questioned; therefore, according to the 5th OCCC and the ESGO/ESMO 2019 consensus conference, a notion of continuum with the “treatment free interval for platinum” has been introduced. Nevertheless, in the present study, we used a dichotomous classification, as this terminology remains widely used in the literature and in clinical practice.

### 4.2. Tissue Microarray/Immunohistochemistry for Estrogen and Progesterone Receptor Expression

In this matched primary/recurrent HGSOC cohort (Table 1), estrogen and progesterone receptor protein detection on tissue microarray slides were performed by staining using a horseradish peroxidase-linked antibody-conjugated automated staining system (Bond Leica Biosystems, Muttenz, Switzerland). For antigen retrieval, paraffin-embedded tissue slides were incubated according to standard procedures. To analyze estrogen receptor expression, slides were incubated with anti-human estrogen receptor-alpha (Clone 6F11, Leica Biosystems, Muttenz, Switzerland, dilution 1:25) for 15 min. For progesterone receptor expression, slides were incubated with monoclonal anti-human progesterone receptor (Clone 16, Leica Biosystems, Muttenz, Switzerland, dilution 1:100) for 15 min. Estrogen and progesterone receptor negative controls involved omission of the primary antibodies. Counterstaining was performed using hematoxylin and 1% acid alcohol. To assess levels of estrogen and progesterone receptor expression, the overall percentage (0–100) and overall intensity (0–3) of each core sample was scored by two independent gynecologic pathologists (S.S. and G.S.). Discrepancies were resolved by consensus. The Immunoreactive Score [42] and the Allred Total score [43] were used, and a total percentage of positive nuclei was recorded. The Immunoreactive Score defines positive estrogen and progesterone receptor expression as a minimum of 10% positive nuclei and moderate to strong staining intensity. Estrogen and progesterone receptor positivity based on the Allred Total score were defined as a minimum of >1% positive nuclei, along with moderate to strong staining intensity. For the data analysis, positive estrogen and progesterone receptor based on the total percentage were defined as staining of >0%.

### 4.3. Statistics

Descriptive statistics were used to analyze demographic data and are summarized as mean ± standard deviation, medians with interquartile ranges or ranges, or frequencies with percentages. Categorical data were compared using the chi-squared or Fisher’s exact test, as appropriate. Paired primary and recurrent HGSOC were compared using two independent immunohistochemistry scores. Percentages in the tables relate to subjects with expression values in primary and relapsed tumors.

To evaluate biomarker discordance in the same patient pre- and post-chemotherapeutic treatments and paired Wilcoxon tests were performed on the Immunoreactive Score and the Allred Total score. To compare biomarker scoring between independent subgroups, unpaired Wilcoxon tests were performed. The results are presented as *p*-values for the corresponding tests.

Statistical significance was set at *p* ≤ 0.05. *p*-values were considered exploratory and were not adjusted for multiple comparisons. Statistical analyses were performed using SPSS software (version 22) and R-version 3.6.3 [44].

## 5. Conclusions

Currently, ovarian cancer has a high relapse rate and a short overall survival. Improved therapeutic approaches, including maintenance therapies, are urgently needed [1]. Our study showed varying levels of estrogen and progesterone receptor expression in primary and paired recurrent tumors, supporting the use of early endocrine maintenance therapy in the first-line setting, following standard chemotherapy for estrogen and/or progesterone receptor positive cancer.

Estrogen and progesterone receptor expression and usage of aromatase inhibitors in HGSOC patients require further scientific attention. Endocrine therapy would substantially improve the quality of life of patients owing to a milder treatment regimen, delayed chemotherapy, and lengthened treatment-free interval for platinum-based chemotherapy. A major challenge of maintenance therapy is to extend the time to next treatment without affecting the quality of life, and it would be extremely useful to prospectively explore the potential of primary endocrine therapy, alone or in combination with other targeted drugs, toward this end.

## Figures and Tables

**Table 1:** Clinicopathological variables of patients with high-grade serous ovarian cancer.

Date of Sampling (Primary and Paired Relapse)	1985–2003
Median age, years - Range	59 20–77
FIGO Stage (at primary diagnosis) - I - II - III and IV	*n* = 80 None 3 (3.7%) 77 (96.3%)
Number of patients, primary chemotherapy-sensitivity - Platinum-sensible - Platinum-resistant	*n* = 80 (100%) 57 (71.3%) 23 (28.7%)
Number of cisplatin-containing cycles - Full = 6 cycles - Incomplete < 6 cycles	*n* = 80 73 (91.3%) 7 (8.7%)

FIGO, Federation of Gynecology and Obstetrics.

**Table 2:** (**a**) Association of estrogen and progesterone receptor immunoexpressions between primary and recurrent tumors (the Immunoreactive Score system). (**b**). Association of estrogen and progesterone receptor immunoexpressions between primary and recurrent tumors (Allred Total Score System).

(a)				
		Primary Tumors	Recurrent Tumors	*p*-Value
Estrogen Receptor	sensitive	12/52 (23.1 %)	9/50 18.0 %	0.74
	resistant	7/21 (33.3 %)	4/21 19.0 %	0.99
	*p*-value	0.57	0.28	
Progesterone Receptor	sensitive	14/48 (29.2 %)	7/52 (13.5 %)	0.030
	resistant	5/21 23.8%	3/20 15.0 %	0.56
	*p*-value	0.84	0.69	
**(b)**				
Estrogen Receptor	sensitive	17/52 (32.7%)	17/50 (34.0 %)	0.84
	resistant	9/21 (42.9%)	11/21 (52.4 %)	0.75
	*p*-value	0.65	0.31	
Progesterone Receptor	sensitive	15/48 (31.3 %)	10/52 (19.2 %)	0.023
	resistant	6/21 (28.6 %)	5/20 (25.0 %)	0.52
	*p*-value	0.73	0.67	

Table 2a shows the association of estrogen and progesterone receptor immunoexpressions (Immunoreactive scoring systems) of primary and recurrent tumors and in chemotherapy (platinum-based)-sensitive and -resistant cases. *p*-values of independent data are based on unpaired Wilcoxon tests on original scores. *p*-values of dependent data are based on paired Wilcoxon tests on original scores. Table 2b shows the association of estrogen receptor and progesterone receptor immunoexpressions (Allred Total Score) of primary and recurrent tumors and in chemotherapy (platinum-based)-sensitive and –resistant cases. *p*-values of independent data are based on unpaired Wilcoxon tests on original scores. *p*-values of dependent data are based on paired Wilcoxon tests on original scores.

## Data Availability

The datasets generated during and/or analysed during the current study are available from the corresponding author on reasonable request.
